# Comparative Analysis of the Impact of Training through Simulation Using the Crisis Resource Management Tool for Primary Care Professionals

**DOI:** 10.3390/healthcare12020230

**Published:** 2024-01-17

**Authors:** Marta Bernardino-Santos, Daniel Arnal-Velasco, Pilar Reboto-Cortés, Cristina Garmendia-Fernandez, Esther Renilla-Sánchez, Ricardo Jose Navalón-Liceras, Elena Botillo-Pérez, Miguel A. Ortega, Juan Ignacio Gómez-Arnau Díaz-Cañabate, Juan A. De León-Luis

**Affiliations:** 1 Department of Anesthesiology and Reanimation, University Hospital Fundación Alcorcón, 28922 Madrid, Spain; marta.bernardino@salud.madrid.org (M.B.-S.); daniel.arnal@salud.madrid.org (D.A.-V.); pilar.reboto@salud.madrid.org (P.R.-C.); ricardojose.navalon@salud.madrid.org (R.J.N.-L.); juangarnau@salud.madrid.org (J.I.G.-A.D.-C.); 2 IDEhA Simulation Center, University Hospital Fundación Alcorcón, 28922 Alcorcon, Spain; elena.botillo@salud.madird.org; 3 Department of Internal Medicine, University Hospital Fundación Alcorcón, 28922 Alcorcon, Spain; cristina.garmendia@salud.madrid.org; 4 Department of Emergency, University Hospital Fundación Alcorcón, 28922 Alcorcon, Spain; mariaesther.renillasanchez@salud.madrid.org; 5 Department of Medicine and Medical Specialities, University of Alcala, 28871 Alcala de Henares, Spain; 6 Ramón y Cajal Institute of Sanitary Research (IRYCIS), 28034 Madrid, Spain; 7 Department of Public and Maternal and Child Health, School of Medicine, Complutense University of Madrid, 28040 Madrid, Spain; jaleon@ucm.es; 8 Health Research Institute Gregorio Marañón, 28009 Madrid, Spain; 9 Department of Obstetrics and Gynecology, University Hospital Gregorio Marañón, 28009 Madrid, Spain

**Keywords:** clinical simulation, experiential learning, medical education, Kirkpatrick scale, crisis resource management, patient safety

## Abstract

This was a prospective observational study based on clinical simulation courses taught in 2017 at the IDEhA Simulation Center of Alcorcón Foundation University Hospital. Two courses in metabolic emergencies (MEs) and respiratory emergencies (REs) were offered to primary care physicians all over Spain. The main objective was to teach nontechnical skills (crisis resource management). Using a modified five-level Kirkpatrick–Phillips education evaluation model, level I (reaction, K1), level II (learning, K2) and level III (behavioral change, K3) changes were evaluated through surveys at the end of the courses and one year later. Thirty courses were held (15 ME courses and 15 RE courses) with 283 primary care physicians. The overall satisfaction (K1) was high: ME courses, 9.5/10; RE courses, 9.6/10. More than 80% of the participants rated the organization, resources, content, debriefing and scenarios as excellent, with no significant differences between the two courses. After one year (156 responses), the respondents for both courses reported that they would repeat the training annually (K2), encourage debriefing with colleagues (K3) and have modified some aspects of their workplace (K3), citing improvements in procedures and in the organization of the health team as the most important. After the ME course, few participants, i.e., 5 (6%), reported providing improved care to patients; after the RE course, 15 (19%) participants reported providing improved care; the difference between groups was significant (*p* < 0.05). Compared with the ME course, the RE course imparted greater knowledge about patient safety (K2) (38 (49%) vs. 24 (31%) (*p* < 0.05)) and more useful tools for daily clinical practice (K3) (67% vs. 56.4%) and resulted in participants paying more attention to personal performance and to colleagues when working as a team (K2) (64% vs. 50%). Clinical simulation courses are highly valued and potentially effective for training primary care physicians in patient safety and CRM tools. Future studies with objective measures of long-term impact, behavior in the workplace (K3) and benefits to patients (K4) are needed. Based on the results of our study, the areas that are important are those aimed at improving procedures and the organization of health teams.

## 1. Introduction

The human factor is defined as the technical skills of health professionals as well as their nontechnical ability to work in a team, communicate effectively, make decisions and handle difficult situations. It has been described as one of the most relevant contributory factors for adverse events, diagnostic errors, medication problems or failures in care processes. The National Study of the Prevalence of Adverse Effects in Primary Care (APEAS) analyzed 96,047 consultations with an observed prevalence of adverse events of 11.2% (95% confidence interval (CI) 10.5–11.9) [1].

One of the strategies described as having the greatest impact on improvements in the human factor is the training of teams through high-fidelity simulation using the Crisis Resource Management (CRM) tool [2].

The analysis of the effect of human factor training through the CRM tool using simulation is referenced in the bibliography for the modified five-level Kirkpatrick–Phillips education evaluation model [3]. The studies were initially carried out in anesthesiology [4] and are being expanded to many other areas, such as obstetrics [5], pediatrics [6] and emergency medicine [7,8]. There are very few specific references to simulation-based training in the area of primary care [9,10,11,12], and those in which CRM training is implemented are out-of-hospital care for individuals with cardiac arrest [13] and trauma [14].

Primary care is an environment of special relevance as a gateway to the health system. On many occasions, primary care requires the management of emergency situations with limited human and technical resources, compared with such resources at hospital emergency departments. The perception of health professionals and patients is that primary care is an area in which patient safety must be reinforced. In some environments, primary care, compared with the work environment in hospitals, is described as fragile due to work overload and, in many cases, difficult and isolated working conditions [15,16,17,18,19].

The objective of this work was to comparatively analyze the impact, based on Kirkpatrick–Phillips levels, of a nontechnical skills training program (CRM) using high-fidelity clinical simulation scenarios between two courses, i.e., metabolic emergencies (MEs) and respiratory emergencies (REs), aimed at primary care physicians throughout Spain.

## 2. Materials and Methods

This was a prospective observational study based on clinical simulation courses taught between January and December 2017 at the IDEhA Simulation Center (Innovation Teaching and Training at the Alcorcón Foundation University Hospital) in Madrid.

The study was presented to and approved by the Ethics Committee of Alcorcón Foundation University Hospital. Prior to the training, the teachers and attendees signed an informed consent form and a confidentiality contract. Everything discussed in the training and in their learning conversations (debriefing) was strictly confidential.

**Figure 1:**
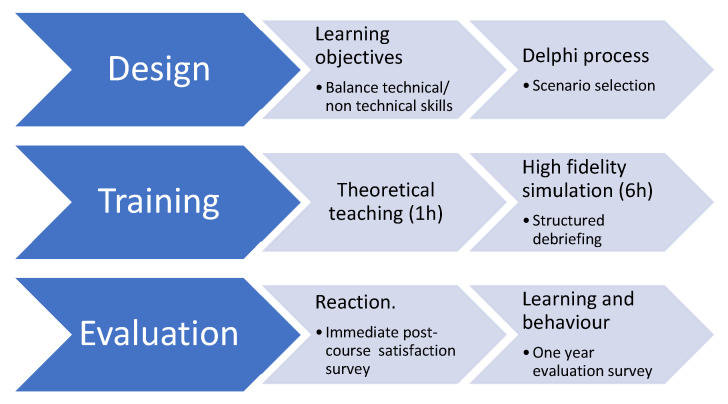
Training program workflow process model.

Two emergency courses for primary care were designed, i.e., metabolic emergencies (MEs) and respiratory emergencies (REs), with four high-fidelity clinical simulation scenarios per course (Figure 1).

The teaching team comprised mixed teams of two specialist doctors and a nurse from the Emergency, Internal Medicine, Anesthesiology and Resuscitation and Surgical departments. The teachers had extensive experience in human factor training and were trained as clinical simulation instructors.

During the second semester of 2016, the teaching team was assembled, a bibliography was compiled and clinical experience, knowledge in patient safety and teaching in simulation were shared. Following the modified Delphi methodology, proposals for simulation scenarios were submitted and evaluated by the entire team. After a second round of consultations and face-to-face meetings, the eight scenarios that simulated urgent clinical situations of metabolic and respiratory diseases with risks to patient safety that primary care physicians could face in their work centers were outlined.

The general objectives of the courses sought to raise awareness, provide information about basic concepts of patient safety and highlight the importance of human factor training in primary care.

The specific objectives of the workshops were to balance the teaching of technical skills (urgent clinical situations of patients with respiratory and metabolic pathologies) with nontechnical skills (key points of the CRM tool).

The courses were aimed at primary care physicians all over Spain, and their attendance was financed by the industry, which did not participate in developing the teaching content of the course. As the courses’ primary objective was to provide continuous education, we made no previous calculations to choose the sample size. Participants were selected based on their availability. The sample size was determined by budget availability.

In the week prior to the courses, the participants received a bibliography for the review consisting of three scientific articles on patient safety education, human factors and CRM [20,21,22].

The maximum number of attendees was defined as 12 per course, and in each of the scenarios, the students were divided into an intervention group (3–4 students) and a mirror group (rest).

Nontechnical skills were common to the ME and RE courses and were selected from the Crisis Resource Management tool [23]:

1. Leadership—common mental model and distribution of workload;
2. Effective communication—assertiveness training (difficult conversations);
3. Help tools in patient transfer processes (handoff);
4. Setting priorities dynamically;
5. Periodic reassessment;
6. Use of cognitive aids;
7. Anticipation and planning—establishing a pause for reflection;
8. Use of all available information and resources.

The courses were eminently practical, with the following time distribution:

Theoretical teaching (1 h)—interactive theoretical seminars aimed at learning the basic concepts of patient safety and the human factor as well as teaching the basic rules of simulation-based training;

High-fidelity simulation exercises (6 h)—four simulation scenarios in a hospital room of the Simulation Center with a high-end adult mannequin (SimMan Essential, Laerdal) with direct viewing and audio from the control room and from the debriefing room for the mirror group that observed the intervening group. The performance of each intervening team was recorded at each stage. The recordings were used to illustrate learning conversations at the discretion of the instructors.

After each scenario, a “structured debriefing” learning conversation was developed in which all students (the intervening group and mirror group) participated [24]. This part of the course was based on an adaptation of Kolb’s method of experiential learning [25]. The debriefing was structured around a review of the *concrete experience* lived during simulation, a *reflective observation* to understand the reasons for the experience, an *abstract conceptualization* of new ideas based on the experience and reflection and, finally, a plan for *active experimentation*, exhorting participants to put into practice the new learned concepts and practices in their day-to-day work. The total or partial videorecorded performance of the intervening participants was viewed as teaching support during the debriefing sessions.

All participants received the training program evaluation surveys. We used the Kirkpatrick–Phillips levels of evaluation [3]. This is an internationally recognized tool for evaluating and analyzing the results of educational, training and learning programs. It consists of five levels of evaluation: reaction, learning, behavioral change, benefits for the patients, and return of investment. Each successive level of the model represents a more precise measure of the effectiveness of a training program.

To evaluate the level of satisfaction of the participants after the course (level I Kirkpatrick, K1) [26], a first evaluation survey (Section S1) was applied to the participants, with ten variables scored using a Likert-type scale (excellent, very good, good, indifferent, bad) related to the content, debriefing, organization, resources, settings and facilities used. At the end of the survey, a global score, from 1 to 10, was obtained for the scenarios, and the participants were asked, through a free-text open question, to provide comments on aspects of the course that could be improved.

To assess the level of learning and impact in the workplace (Kirkpatrick levels II and III, respectively, K2 and K3) [26], one year after the completion of all courses, a second evaluation survey (Section S2) was sent to all participants. This second survey included five variables scored using a Likert-type scale from 1 (completely disagree) to 5 (completely agree) and two variables with a YES/NO answer option. The variables “increase in knowledge about patient safety”, “useful tools for daily practice”, “pay more attention to personal and team performance” and “repeat training annually” assess learning (K2). The variables “carrying out debriefing with colleagues after crisis situations”, “modification of some aspect in the workplace” and “modification of health care aspects with patients” assess behavioral change (K3) in the workplace.

The variable about the aspects modified in the workplace after completing the course was followed by a conditional question: if the response was affirmative, a second closed-answer question was shown to address five aspects (improvement in procedures, organization of the health team, changes in the organization, changes in the workplace or other). These five aspects are usually analyzed as triggers in the analysis of adverse events associated with health care [27].

The evaluation consisted of two anonymous surveys: one paper-based survey distributed at the end of the training session and a second online formulary survey (https://es.surveymonkey.com/) sent through email one year following the session.

The data collected from both surveys were stored in an Excel database divided by course profile (ME and RE) by an independent administrative staff.

In the first survey (K1), the responses scored as excellent were grouped together with the responses scored as not excellent (very good, good, indifferent and bad). In the second survey (K2 and K3), the responses scored as favorable, 4 or 5 (totally agree and agree), were grouped, as were the unfavorable ones, 3, 2 and 1 (indifferent, disagree, and totally disagree).

For the statistical analysis of the quantitative variables, the means of both groups were compared, and the statistical significance was analyzed by means of the Student’s t test. For comparative analyses of percentages using the chi-square test, statistical significance was tested for each variable in each survey. *p* < 0.05 was used as the cutoff point for statistical significance.

## 3. Results

A total of 30 sessions of the CRM courses were held: 15 sessions of the ME course and 15 sessions of the RE course. A total of 283 students were trained, all primary care physicians from 16 of the 17 autonomous communities in Spain. Of these, 138 (48.8%) students took the ME course, and 145 (51.2%) took the RE course. There were an average of 9.2 students in the ME course and 9.6 students in the RE course.

In the first survey (K1) of satisfaction after the course, the overall scores for the courses were 9.5 and 9.6 out of 10 for the ME and RE courses, respectively. Table 1 provides the percentage of excellent vs. not excellent responses (very good, good, indifferent, and poor) of the first survey for all students in each course (ME/RE), as well as the Delta (difference between the two groups) for each of the variables studied. More than 80% of the students rated the organization, resources, content, debriefing, usefulness and simulation scenarios as excellent in both courses (ME/RE). There were no significant differences in the evaluations between courses.

Of the 283 surveys sent a year later, in which learning (K2, variables 1, 2, 3 and 4) and behavioral change in the workplace (K3, variables 5, 6 and 7) were evaluated, we received responses from 156 students (55%), i.e., 78 (50%) from the ME course and 78 (50%) from the RE course.

Table 2 provides the response percentages for variables 1, 2, 3, 4 and 5 based on a score (1–5), and for variables 6 and 7, the percentages of YES/NO responses are provided.

**Figure 2:**
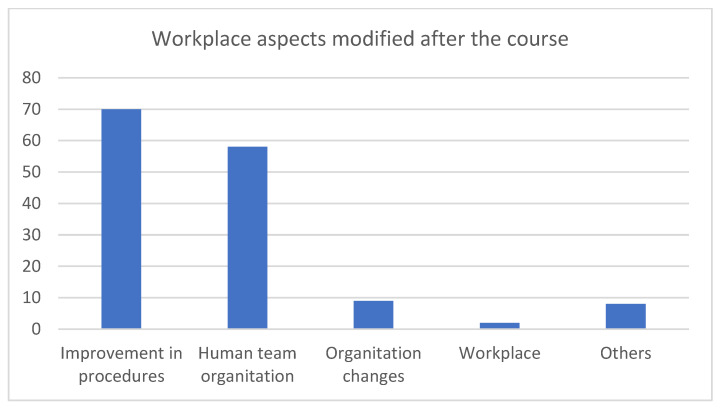
Workplace aspects modified after the course (survey 2, variable 6).

Table 3 provides the responses with favorable scores (4 or 5) by course (ME/RE). Table 4 shows the comparative analysis of the responses with unfavorable scores (1, 2 or 3) by course (ME/RE). Figure 2 presents aspects of the workplace that were modified after the course (variable 5).

For knowledge about patient safety (K2), there were significant differences between the courses; specifically, students who completed the ME course reported lower levels of knowledge about patient safety than students who completed the RE course (38 (49%) vs. 24 (31%)) (*p* < 0.05) (Table 4). Despite not finding significant differences, there was a greater tendency among the students who completed the RE course than among the students who completed the ME course to consider that the course provided them with useful tools for daily clinical practice (K2) (67% vs. 56.4%) and caused them to pay more attention to personal performance and teamwork (K2) (64% vs. 50%). In both courses, almost all the students (99% and 97%) responded favorably to the possibility of being able to repeat simulation-based training on an annual basis (K2).

For both course profiles, the majority of the students responded that they encouraged more debriefing with their colleagues after joint work in a crisis situation (K3) (79.5% vs. 74.4%) and that the course has led to modifications in some aspects of normal work (K3) (94% and 91%) (Table 3), citing improvements in procedures and in the organization of health teams as the most important (Figure 1). Finally, there were significant differences between the courses in terms of the percentage of students who reported no improvements in the care of their patients after the course; this percentage was higher among the students who completed the ME course than among those who completed the RE course (15 (19%) vs. UR 5 (6%)) (*p* < 0.05) (Table 4).

## 4. Discussion

To our knowledge, this is the first study that analyzes the impact of 30 courses offered nationally to primary care physicians (283) in Spain that focused on improving the human factor in crisis situations. The primary result was that the courses significantly improved student knowledge and led to modifications in work environments one year after completing the courses.

In this study, for the two high-fidelity simulation courses (ME/RE), the overall satisfaction of the students with the courses was notable, i.e., greater than 9.5 out of 10, with more than 80% being responses of “excellent”, without significant differences between the two courses.

In the opinion of the students, the impact of the courses one year after completion included increased knowledge about patient safety, the acquisition of useful tools for daily practice, the ability to pay more attention to personal and team performance and the desire to repeat such trainings annually. Regarding those who reported that some aspects of their work environment had been modified (>90% of those who took the ME and RE courses), the majority reported improvements in procedures and in the organization of health teams. If these reported data are confirmed, the training activity would reach level 3 on the Kirkpatrick scale.

Primary care professionals should receive training in patient safety throughout their professional career, training that is adapted to their environment and involves patients and work teams [18,28,29,30].

The studies published on training projects aimed at primary care describe training with simulated patients in telephone consultations and virtual reality, and most are aimed at resident physicians [9,10,11,12]. A recent review found 49 simulation-based training studies for different learning objectives and methodologies in primary care [31]. The design of a course aimed specifically at experienced physicians (nonresidents in training) based on simulation scenarios is novel and aims to transmit and put into practice concepts of patient safety and CRM tools.

The teaching of CRM based on experiential models with simulation, in which participants believe that the knowledge acquired will improve their future competencies [32], seems to be one of the best learning methods for adults. The results of our study corroborate this.

We decided to use Kirkpatrick’s five-level model to measure the impact of our training program, as it grades the evidence from a simple initial reaction of the attendants to actually improving patient outcomes and eventually the return on investment [26].

According to Kirkpatrick’s five-level model, level one (K1) measures the satisfaction (reaction) described by participants after an intervention [26]. In our study, it is notable that these high-fidelity simulation courses received such high satisfaction scores, given that the courses were completed by numerous primary care physicians throughout the national territory. Furthermore, the fact that there are no differences between the two course profiles suggests that both courses are equally effective in terms of student reaction.

One of the most relevant data points, according to the opinion of the students one year after having completed the courses, is that both courses contributed to increasing their knowledge about patient safety by providing them with useful tools in their daily practice and by causing them to pay more attention to their personal and team performance. Our results reflect learning (K2) in terms of the acquisition of competencies or abilities and, more noteworthy, one year later, self-perceived behavioral change in their primary care workplace (K3). In previous primary care simulation-based training studies, efficacy was also evaluated across the Kirkpatrick levels and demonstrated a positive impact for knowledge-, skills- and attitude-based outcomes, though this was limited in select studies [31]. None tried to address a K3 proxy. Although individual perception has limitations, learning is described as a reflection of the acquisition of skills after receiving training [32,33]. In similar studies that describe results one year after training activities in an anesthesia environment and more recently in obstetrics, the findings are consistent with ours regarding the acquisition of useful skills for daily practice and communication with their team [34,35]. In these studies, the participants were part of work teams that included not only physicians of several specialties but also residents. As in our study, the repetition of training actions and debriefing with classmates are recommended [35]. As potential reasons for the reported success of our training program, we hypothesize the debriefing structure, using Kolb’s method of experiential learning [25], added to the strong emotional experience recreated by the high-fidelity scenarios, and the explicit commitment gathering to take the new practices back to daily work could have played a role in these results. Other favorable conditions were the high tutor experience, tutor–student rate, and small number of participants per session, which permitted deep reflective discussions and individual needs tailoring. Noteworthy, the reported impact was achieved through a single training session.

Finally, regarding the behavioral change in the workplace (K3), the students who completed the ME course reported fewer modifications to the workplace and patient care than those who completed the RE course. Considering that the teaching team and the nontechnical skills teaching part (CRM) were common to both courses, the clinical portion of the courses explains these differences. Metabolic emergencies are less common than respiratory emergencies in primary care clinics; therefore, their clinical applicability may have been lower and may have influenced this result.

We present the results of a simulation-based training project that uses scenarios specifically designed for primary care physicians at the national level; a follow-up occurred immediately and one year after completing the courses. The entire teaching team, apart from their extensive clinical experience, had the same qualifications as the clinical simulation instructors, allowing for a homogenous methodology during the course and *debriefing* after the scenarios for all the course sessions.

The response rate for the survey applied immediately after the course was high; twelve months later, 55% of the participants responded to the follow-up survey. As positive aspects that contributed to this good participation, the digital questionnaire was short and easy to answer, and a personalized message was sent to the students by the teachers to remind them about the training they received.

Participation could be further improved by sending follow-up surveys after 6 months and providing reminders, e.g., via telephone, that the survey is available for completion.

The group of students who completed the courses only included primary care physicians, not multiprofessional teams. To reinforce the impact in the workplace, it would be of great value to promote courses aimed at multiprofessional teams and consider alternative teaching strategies when resources are more limited, such as simulation in mixed distance and face-to-face models “in situ” with lower-cost mannequins [36].

Due to the anonymous characteristics of the evaluation surveys, we could not track the respondents’ demographic data, such as gender, years of practice or type of primary care facility, that might influence the responses.

A final limitation of this study is the measure of learning (K2) and behavioral change (K3) based on a learning self-reported perception. While we did not have an objective measure in the workplace before and after completing the course, self-assessment has been suggested as a valid tool to assess the acquisition of skills [33] and provides a reasonable feasibility balance in terms of resources needed to evaluate the training programs. One-year self-reflected behavioral change in the workplace may be included in future training programs and policy evaluations as a K3 proxy (Figure 1).

## 5. Conclusions

In general, these results suggest that high-fidelity simulation courses are a well-valued and possibly effective strategy for the training of and continuous improvement of primary care physicians in terms of knowledge and tools to improve patient safety.

The students of both courses reported, without differences between the two courses, high satisfaction with the courses from which they learned and gained skills that they implemented in their daily practice. Those who completed the RE course reported a greater knowledge of patient safety and a greater occurrence of modifications to their health care practices.

Future studies are needed to investigate work teams in primary care through objective measures of the long-term impact of behavior in the workplace (Kirkpatrick level 3), benefits to patients (Kirkpatrick level 4) and eventually return on investment (Kirkpatrick level 5). Based on the results of our study, the areas that are most important are those that aim to improve procedures and the organization of health teams.

## Figures and Tables

**Table 1:** Comparative analysis of the grouped responses (excellent) for each of the variables in the first survey by course (ME/RE).

Workshop-Related Variables	Total (n = 283)	ME (n = 138)	RE (n = 145)	Delta	*p*
Organization	87.63	89.86	85.52	4.34	0.27
Facilities used	78.45	77.54	79.31	−1.77	0.7
Available resources	81.27	81.16	81.38	−0.22	0.9
Comfort of the classroom	57.95	55.80	60.00	−4.20	0.5
Duration of each scenario	68.90	68.12	69.66	−1.54	0.8
Number of students in each scenario	77.39	78.99	75.86	3.12	0.5
Content level	82.33	83.33	81.38	1.95	0.3
Post-stage debriefing	86.22	84.78	87.59	−2.80	0.5
Usefulness of the content learned	84.10	83.33	84.83	−1.49	0.1
General impression of the scenarios	83.39	81.88	84.83	−2.94	0.5

**Table 2:** Responses to the second follow-up survey sent one year after the courses were taught.

Survey Responses (One Year after Courses, 2018) (n = 156)					
	Strongly Agree (5)	4	3	2	Strongly Disagree (1)
After finishing this course, I felt that my knowledge about patient safety had increased. (K2)	94 (60.3%)	61 (39.1%)	0	0	1 (0.6%)
2. The course has provided me with useful tools for my daily clinical practice. (K2)	96 (61.5%)	56 (35.9%)	3 (1.9%)	0	1 (0.6%)
3. After participating in the course, I pay more attention to my performance and that of my colleagues when we work as a team. (K2)	89 (57.05%)	65 (41.7%)	1 (0.6%)	0	1 (0.6%)
4. I would repeat simulation training with my work team on an annual basis. (K2)	120 (76.9%)	33 (21.1%)	1 (0.6%)	1 (0.6%)	1 (0.64%)
5. I am encouraging *debriefing* among my coworkers after working together in a crisis situation. (K3)	43 (27.6%)	77 (49.4%)	24 (15.4%)	11	1 (0.6%)
	**yes**	**no**			
6. The course has modified some aspect of my usual work. (K3)	144 (92.3%)	12 (7.7%)			
7. The course has improved some aspect of my care of patients. (K3)	136 (87.2%)	20 (12.8%)			

**Table 3:** Comparative analysis of the percentage of favorable responses (score 4 or 5) by course (ME/RE) in the second follow-up survey.

	Total	ME	RE	*p*
After finishing this course, I felt that my knowledge about patient safety had increased. (K2)	99.4	98.7	100	0.32
2. The course has provided me with useful tools for my daily clinical practice. (K2)	97.7	98.7	96.2	0.31
3. After participating in the course, I pay more attention to my performance and that of my colleagues when we work as a team. (K2)	98.7	98.7	98.7	1
4. I would repeat simulation training with my work team on an annual basis. (K2)	98.1	98.7	97.4	0.56
5. I am encouraging *debriefing* among my coworkers after working together in a crisis situation. (K3)	76.9	74.4	79.5	0.45
6. The course has modified some aspect of my usual work. (K3)	92.3	91	93.6	0.55
7. The course has improved some aspect of my care of patients (K3)	87.2	80.8	93.6	0.02

**Table 4:** Comparative analysis of the percentage of unfavorable responses (score 1, 2 or 3) by course (ME/RE) in the follow-up survey.

	Total	UM	UR	*p*
After completing this course I felt that I had increased my knowledge about patient safety. (K2)	62 (39.7%)	38 (48.7%)	24 (30.8)	0.02
2. The course has provided me with useful tools for my daily clinical practice. (K2)	60 (38.5%)	34 (43.6%)	26 (38.5%)	0.2
3. After participating in the course, I pay more attention to my performance and that of my colleagues when we work as a team. (K2)	67 (42.9%)	39 (50%)	28 (35.9%)	0.07
4. I would repeat simulation training with my work team on an annual basis. (K2)	36 (23.1%)	18 (23.1%)	18 (23.1%)	1
5. I am encouraging *debriefing* among my coworkers after working together in a crisis situation. (K3)	113 (72.4%)	52 (66.7%)	61 (78.2%)	0.1
6. The course has NOT modified any aspect of my usual work. (K3)	12 (7.7%)	7 (9%)	5 (6.4%)	0.5
7. The course has NOT improved any aspect of my care of patients. (K3)	20 (12.8%)	15 (19.2%)	5 (6.4%)	0.02

## Data Availability

The data used to support the findings of the present study are available from the corresponding author upon request.

## Supplementary Materials

The following supporting information can be downloaded at: https://www.mdpi.com/article/10.3390/healthcare12020230/s1, Section S1. First Survey at the End of the Course; Section S2. Second Survey Sent One Year after Completing the Course.

## References

[1] Ministerio de Sanidad y Consumo (2008). Estudio APEAS. Estudio Sobre La Seguridad De Los Pacientes En Atención Primaria De Salud.

[2] Buljac-Samardžić M., Doorn C.M.D.-V., Maynard M.T. (2021). What Do We Really Know About Crew Resource Management in Healthcare? An Umbrella Review on Crew Resource Management and Its Effectiveness. J. Patient Saf..

[3] Phillips J. Return on investment—Beyond the four levels. Proceedings of the Academy of Human Resource Development Conference.

[4] Gaba D.M., Howard S.K., Fish K.J., Smith B.E., Sowb Y.A. (2001). Simulation-Based Training in Anesthesia Crisis Resource Management (ACRM): A Decade of Experience. Simul. Gaming.

[5] Bracco F., De Tonetti G., Masini M., Passarelli M., Geretto F., Celleno D. (2018). Crisis Resource Management in the Delivery Room: Development of Behavioral Markers for Team Performance in Emergency Simulation. Int. J. Environ. Res. Public Health.

[6] Bank I., Snell L., Bhanji F. (2014). Pediatric Crisis Resource Management Training Improves Emergency Medicine Trainees’ Perceived Ability to Manage Emergencies and Ability to Identify Teamwork Errors. Pediatr. Emerg. Care.

[7] Reznek M., Smith-Coggins R., Howard S., Kiran K., Harter P., Sowb Y., Gaba D., Krummel T. (2003). Emergency Medicine Crisis Resource Management (EMCRM): Pilot Study of a Simulation-based Crisis Management Course for Emergency Medicine. Acad. Emerg. Med..

[8] Angulo C.C., María Quintillá Martínez J., Espinosa Ramírez S. (2020). Los Orígenes: Crew Resource Management Emergency Crisis Resource Management SEMES (E-CRM SEMES) Clinical Simulations and Safety in Emergencies: Emergency Crisis Resource Management. Emergencias.

[9] Gilic F., Schultz K., Sempowski I., Blagojevic A. (2019). “Nightmares–Family Medicine” Course Is an Effective Acute Care Teaching Tool for Family Medicine Residents. Simul. Healthc..

[10] Beddows J., Abdalla M., Blanchard D., Hammond E., Hay F., Webb M., Protheroe J. (2021). ‘I’m just ringing to get a repeat prescription for my contraceptive pill, doctor’: Developing authentic simulated telephone consultations for medical students. Educ. Prim. Care.

[11] Morreel S., Philips H., Colliers A., Verhoeven V. (2020). Performance of a new guideline for telephone triage in out-of-hours services in Belgium: A pilot study using simulated patients. Health Serv. Manag. Res..

[12] Pan X., Slater M., Beacco A., Navarro X., Rivas A.I.B., Swapp D., Hale J., Forbes P.A.G., Denvir C., Hamilton A.F.d.C. (2016). The Responses of Medical General Practitioners to Unreasonable Patient Demand for Antibiotics—A Study of Medical Ethics Using Immersive Virtual Reality. PLoS ONE.

[13] Castelao E.F., Boos M., Ringer C., Eich C., Russo S.G. (2015). Effect of CRM team leader training on team performance and leadership behavior in simulated cardiac arrest scenarios: A prospective, randomized, controlled study. BMC Med. Educ..

[14] Johnson G.G., Beaumont J., Paton-Gay J.D., Widder S., Gillman L.M. (2021). Multidisciplinary, multisite trauma team training during COVID-19: Lessons from the first virtual E-S.T.A.R.T.T. course. Can. J. Surg..

[15] Curran C., Lydon S., E Kelly M., Murphy A.W., O’connor P. (2019). An analysis of general practitioners’ perspectives on patient safety incidents using critical incident technique interviews. Fam. Pract..

[16] de Wet C., Bowie P., O’donnell C. (2018). ‘The big buzz’: A qualitative study of how safe care is perceived, understood and improved in general practice. BMC Fam. Pract..

[17] Harbitz M.B., Stensland P.S., Gaski M. (2022). Rural general practice staff experiences of patient safety incidents and low quality of care in Norway: An interview study. Fam. Pract..

[18] Madden C., Lydon S., Murphy A.W., O’Connor P. (2022). The patient’s “story”: An examination of patient-reported safety incidents in general practice. Fam. Pract..

[19] Rodríguez-Cogollo R., Paredes-Alvarado I.R., Galicia-Flores T., Barrasa-Villar J.I., Castán-Ruiz S. (2014). Cultura de seguridad del paciente en residentes de medicina familiar y comunitaria de Aragón. Rev. Calid. Asist..

[20] Rall M., Gaba M., Miller R.D. (2005). Human Performance and Patient Safety. Miller’s Anesthesia.

[21] Rall M., van Gessel E., Staender S. (2011). Education, teaching & training in patient safety. Best Pract. Res. Clin. Anaesthesiol..

[22] Reason J. (1995). Understanding adverse events: Human factors. Heart.

[23] Gaba D.M. (2010). Crisis resource management and teamwork training in anaesthesia. Br. J. Anaesth..

[24] Rudolph J.W., Simon R., Rivard P., Dufresne R.L., Raemer D.B. (2007). Debriefing with Good Judgment: Combining Rigor-ous Feedback with Genuine Inquiry. Anesthesiol. Clin..

[25] Kolb D.A. (1984). Experiential Learning: Experience as the Source of Learning and Development.

[26] Kirkpatrick D.L. (1998). The Four Levels of Evaluation. Evaluating Corporate Training: Models and Issues.

[27] Bartolomé A., Gómez-Arnau J.I., del Valle S.G., González-Arévalo A., Santa-Úrsula J.A., Hidalgo I. (2005). Seguridad del paciente y sistemas de comunicación de incidentes. Rev. Calid. Asist..

[28] Spencer R., Campbell S.M. (2014). Tools for primary care patient safety: A narrative review. BMC Fam. Pract..

[29] Portela Romero M., Bugarín González R., Rodríguez Calvo M.S. (2017). La cultura de seguridad del paciente en los médicos internos residentes de Medicina Familiar y Comunitaria de Galicia. Atención Primaria.

[30] Campbell S.M., Bell B.G., Marsden K., Spencer R.B.B., Kadam U., Perryman K., Rodgers S., Litchfield I., Reeves D., Chuter A.B. (2018). A Patient Safety Toolkit for Family Practices. J. Patient Saf..

[31] Bray L., Krogh T.B., Østergaard D. (2023). Simulation-based training for continuing professional development within a primary care context: A systematic review. Educ. Prim. Care.

[32] Østergaard D., Dieckmann P., Lippert A. (2011). Simulation and CRM. Best Pract. Res. Clin. Anaesthesiol..

[33] Crofts J., Ellis D., Draycott T., Winter C., Hunt L., Akande V. (2007). Change in knowledge of midwives and obstetricians following obstetric emergency training: A randomised controlled trial of local hospital, simulation centre and teamwork training. BJOG Int. J. Obstet. Gynaecol..

[34] Blum R.H., Raemer D.B., Carroll J.S., Sunder N., Felstein D.M., Cooper J.B. (2004). Crisis resource management training for an anaesthesia faculty: A new approach to continuing education. Med. Educ..

[35] Gardner R., Walzer T.B., Simon R., Raemer D.B. (2008). Obstetric Simulation as a Risk Control Strategy: Course design and evaluation. Simul. Healthc..

[36] Schnaubelt S., Garg R., Atiq H., Baig N., Bernardino M., Bigham B., Dickson S., Geduld H., Al-Hilali Z., Karki S. (2023). Cardiopulmonary resuscitation in low-resource settings: A statement by the International Liaison Committee on Resuscitation, supported by the AFEM, EUSEM, IFEM, and IFRC. Lancet Glob. Health.

