# Supplementary material for: Comparative Analysis of the Impact of Training through Simulation Using the Crisis Resource Management Tool for Primary Care Professionals
Source: Healthcare (Basel). 2024 Jan 17;12(2):230. doi: 10.3390/healthcare12020230 (PMC10815590; doi:10.3390/healthcare12020230)
Supplement: Supplementary file 1 [file healthcare-12-00230-s001.zip › healthcare-2757026-supplementary.pdf]

Supplementary Materials

Section S1. First Survey at the End of the Course

Name of the course: **PATIENT SAFETY IN RESPIRATORY/METABOLIC EMERGENCIES IN PRIMARY CARE**

Course date:

	EXCELLENT	VERY GOOD	Good	INDIFFERENT	Bad
Organization					
Facilities used					
Available resources					
Comfort of the classroom					
Duration of each scenario					
Number of students in each scenario					
Content level					
Post-stage debriefing					
Usefulness of the content learned					
General impression of the scenarios					

Please rate the simulation scenarios with a score of 0 to 10 points:

If **we have missed something or you want to propose something, please use the space below to comment:**

Section S2. Second Survey Sent One Year after Completing the Course
The course was as follows:

Respiratory Emergencies OR Metabolic Emergencies

Indicate your level of agreement-disagreement with the following statements

Strongly agree 5 4 3 2 1 Completely disagree

1. After completing this course, I felt that my knowledge about patient safety had increased.
5 4 3 2 1

2. The course has provided me with useful tools for my daily clinical practice
5 4 3 2 1

3. After participating in the course, I pay more attention to my performance and that of my colleagues when we work as a team
5 4 3 2 1

4. I would repeat simulation training with my work team on an annual basis
5 4 3 2 1

5. I am encouraging debriefing among my coworkers after working together in a crisis situation.
5 4 3 2 1

6. The course has modified some aspect of my usual work

YES/NO

Please indicate which:

- **Improvement in procedures**
- **Organization of the health team**
- **Place of work**
- **Changes in the organization**

Other ……………………….

7. The course has improved some aspect of my care of patients
YES/NO

Please indicate which ________________________
